# Designing an Informal Payment Model for Patients Admitted in the Iran Health System: A qualitative Study

**DOI:** 10.4314/ejhs.v31i4.24

**Published:** 2021-07

**Authors:** Esmaeil Rezazadeh, Ghahraman Mahmoudi, Fatemeh Dabaghi

**Affiliations:** 1 Student of Ph.D. by Research, Hospital Administration Research Center, Sari Branch, Islamic Azad University, Sari, Iran; 2 Associated Professor of Hospital Administration Research Center, Sari Branch, Islamic Azad University, Sari, Iran; 3 Associated Professor of Hospital Administration Research Center, Sari Branch, Islamic Azad University, Sari, Iran

**Keywords:** Informal Payment, Model design, Hospitalized patients, Health System, Qualitative study

## Abstract

**Background:**

Countries rely on out-of-pocket spending to different degrees and employ varying techniques. This study aimed at designing an out-of-pocket payment model of patients admitted to the Iran health system.

**Methods:**

This study was a combined qualitative and quantitative study. The statistical population of the qualitative section was 30 experts who were purposefully selected and continued by snowball method until data saturation, and in the quantitative section were 212 managers of the Ministry of Health. The questionnaire was designed based on qualitative analysis. Content validity was confirmed based on expert opinion and structural validity using exploratory factor analysis and confirmatory factor analysis. Reliability was confirmed using CRONBACH's alpha coefficient and composite reliability. For model adequacy, KMO index and Bartlett test were used, and for model fit, CFI and IFI fitness index were used.

**Results:**

Based on the results of 6 main themes, 20 Concept and 120 sub-themes of out of pocket payment of hospitalized patients were extracted. The value of chi-square was 4599.861, the degree of freedom was 2421 and the result of their ratio was 1.899 in the model, which was an acceptable value. CFI and IFI fitness indicators are acceptable. The SRMR index was 0.1153, which indicates the adequacy of the model.

**Conclusion:**

The findings showed that the main dimensions of out of pocket payment of hospitalized patients include causal factors, underlying factors, intervening factors, pivotal categories, strategies and consequences. Therefore, the use of a paradigm model to pay attention to all the effective dimensions in reducing the payment of hospitalized patients is recommended.

## Introduction

Good health system is an essential requirement for sustained economic, social development and decreasing the rate of poverty, but people need to be protected from being forced into poverty as a result of the cost of health care (1). Health financing is critical for reaching Universal Health Coverage (UHC), but nowadays the financing mechanism, and also finding some ways for providing resources, repaying costs, and improving insurance coverage of the community have become major challenges in many health systems (2). At the timed that a government is unable to properly finance healthcare system, the burden of funding will directly affect the people, and they have to pay for health services by themselves (3,4). Direct payments made by individuals to health care providers at the time of service use is known as out-of-pocket payments (OOPs), which is the weakest and most unfair payment mechanism in health care system (5).

Out-of-pocket payments are one of the most common sources of health systems financing around the world, in which the patient pays the cost of purchasing and consuming services and goods in cash. OOPs are not reimbursable by insurers or third parties (6). And, include official co-payments, and informal payments (7).

Informal payments are illegal and often a form of corruption (8). Patients or their relatives make these payments to health care providers out of the official bills (9). Informal payments have the following characteristics: a) not registered; b) not necessarily related to treatment; c) paid before, during, and after the treatment process; and d) may be initiated by the hospital staff or patient (8). In addition, these payments are made in cash or non-cash form (10).

Informal patient payments for health services are common in many health systems, especially in developing countries (11). Evidence indicates that these payments occur in at least 22 African, Asian and European countries (12). Information on the extent of informal payments is negligible due to their illegal nature, but studies have shown that between 10% and 45% of total health expenditures in some low-income countries relates to this type of payment (13). Previous studies in Iran have reported different frequency of informal payments in the health system where amounts of informal payments ranged from 5.93% to 63.8% (14–18). Recently, the findings of a systematic review showed that the amount of informal payments was around 35% (19). Lack of public resources to finance health services, lack of adequate monitoring and supervision, poor complaints handling, low salaries and benefits to health care providers, poor management, delay of insurance companies in reimbursing costs, unreliability of tariffs and socio-cultural characteristics are among the factors contributing to informal payments (10,15).

Evidence suggests that informal payments have a negative impact on access and use of healthcare, equity, efficiency, quality, and patient-doctor relationship (20). These payments increase the cost of care for patients, especially the poor people, and cause some people to discontinue using health services or treatment process (21).

There are several strategies to decrease negative outcomes of informal payments, such as to increase rewards to providers, to increase the government's share of health expenditures, to put emphasis on the rules and punishment for flouting them, as well as to bring about behavior change (22). And, one of its goals is to reduce and eliminate informal payments in health system (23). In this regard, the government is committed to reducing informal payments by increasing medical tariffs, increasing its own share in health system financing and expanding insurance coverage (18).

Earlier studies proved that one of the drawbacks of Iran's health system is unofficial payments (13,18, 24). The ‘Health Transformation Plan (HTP) since 2014, with a particular focus on financial protection. This plan consists of eight service packages, and one of its main goals is to reduce IPs or eliminate it from the health system that cause serious barriers in health system reform (23). In 2019, about 27.7% of Iranian population had at least one IPs experience for health services and the prevalence rates of compulsory and voluntary IPs were 21.4 and 11.5%, respectively. In addition, IPS were Reported to be 26.1% in inpatient wards (13).

It was expected that the implementation of this reform would reduce the frequency of informal payments. Progress in implementing reforms and the extent to which the goals have been achieved should be monitored and evaluated regularly. Due to the importance of the subject, the aim of this study was to design an informal payments model for inpatients in order to provide feedbacks to policy makers in the Iranian health system.

## Material and Methods

This study was a combined qualitative and quantitative study, which was conducted in 2020, purposive sampling was used to select the participants. Those who have at least 10 years of experience in policy and planning in the Iran health system were included in the study. The Interview lasted 30 to 45 minutes in a quiet environment. The interview method was done via Skype, telephone service and online video. At the beginning of each interview, the research objectives for the participant were explained, and with their permission, the interview was recorded. Also, in order to observe the ethical issues, participation in the study was optional and the confidentiality of the information was guaranteed. The study population consisted of 30 experts who were purposefully selected and continued by snowball method until data saturation and in quantitative was included 212 experts of the Ministry of Health. In collecting qualitative data, a semi-structured interview guide was used. The interview guide based on the paradigm model of Strauss and Corbin had 5 questions about informal payment of patients admitted to the world health system. The validity of the questionnaire was confirmed by 5 experts.

The questionnaire based on qualitative analysis was designed. The dimensions of informal payment of patients admitted were determined based on five steps. The items to prepare a questionnaire for the quantitative stage were designed. The questionnaire consisted of 69 questions 5-choice on the LIKERT scale, the validity of which in terms of formal and content was formalized, which finally was confirmed. Content validity was confirmed based on expert opinion and structural validity using exploratory factor analysis and confirmatory factor analysis. Reliability was confirmed using CRONBACH's alpha coefficient and composite reliability. For model adequacy, KMO index and Bartlett test were used, and for model fit, CFI and IFI fitness index were used.

Foundation data theory method was used to analyze the data obtained from the interviews. Research data using three types of coding; open, axial and selective coding were analyzed. Research categories were explored and identified through open coding through concepts. Then, in axial coding, the axial category was identified and the relationship between this category and subcategories was illustrated using the Strauss and Corbin paradigm. Then, in axial coding, the axial category was identified and the relationship between this category and subcategories was depicted using the Strauss and Corbin paradigm. Finally, in the selective coding, a coherent narrative was presented using the obtained categories and their characteristics and dimensions. In this section, the accuracy and precision of the identified features were confirmed through a specialized panel. Data analysis was performed using MAXQDA10 software.

The present study with the code: ID IR.IAU.CHALUS.REC.2018.019 has been approved by the ethics committee of Islamic Azad University and the necessary ethical points have been observed in this research. At the beginning of the interview, participants were introduced to the purpose of the study and provided full informed consent to participate in the study.

## Results

This study was conducted to design an informal payment of patients admitted to the Iran health system. By extracting the codes, the model was classified and designed in the form of 120 basic codes, 20 concepts and 6 main categories of causal factors, contextual factors, central category (concept of payment rate reduction), intervening conditions, strategies and consequences. This section refers to the code formation by mentioning the quotes of the interviewees.

**Figure 1:**
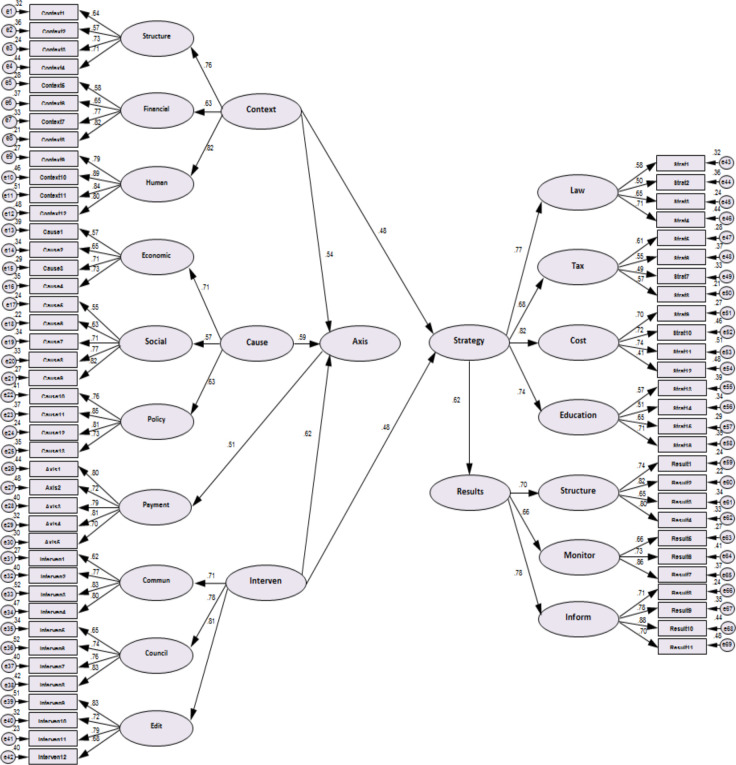
The proposed model in the mode of standard coefficients

According to the codes, the proposed drawing model for control of informal payments of inpatients is presented in the Figure 1.

As can be seen in Table 2, the standard factor coefficient of causal conditions in the axial phenomenon (informal payment of patients admitted) is equal to 0.59, t-value is equal to 7.214 and P-value is less than 0.05. As a result, from the perspective of those present in the study, causal factors are effective in informal payment control of patients admitted.

**Table 2:** Investigating the relationship between the variables of the paradigm model of informal of payment of hospitalized patients

Results	P-Value	Amounts t	Standard coefficients	Relationship between paradigm model variables
It is meaningful	< 0/0001	7/214	0/59	Axial category ← Causal factor
It is meaningful	< 0/0001	7/685	0/54	Axial category ← Underlying factors
It is meaningful	< 0/0001	9/121	0/62	Axial category ← interfering factors
It is meaningful	< 0/0001	6/714	0/48	Strategies ← Underlying factors
It is meaningful	< 0/0001	9/121	0/62	consequences ← Strategies

The standard factor coefficient of the background condition variable in the axial phenomenon (informal payment of inpatient) is equal to 0.54, t value is equal to 7.685 and the value of P-value is less than 0.05. Participants in the study, the underlying factors are effective in informal the payment control of patients admitted.

The standard factor coefficient of the intervening condition variable in the axial phenomenon (informal the payment of patients admitted) is equal to 0.69, t-value is equal to 9.121 and the value of P-value is less than 0.05. In the research, the interfering factors are effective in informal the payment control of patients admitted.

The standard coefficient of variable of underlying factors in the strategy is equal to 0.48, t value is equal to 6.714 and the value of P-value is less than 0.05. The conclusion is that, from the point of view of the participants in the research, the underlying factors in the adopted strategy are effective.

The standard factor coefficient of the strategy variable adopted in the consequences is equal to 0.62, t value is equal to 9.121 and the value of P-Value is less than 0.05. The conclusion is that, from the perspective of those present in the study, the strategy adopted is effective in outcome.

**The degree of appropriateness of the final model of informal the payment of hospitalized patients**

As can be seen in Table 3, the value of the chi-square statistic in the model 165/4599, the degree of freedom of the model is equal to 2421, the result of their ratio is equal to 1.899, which is an acceptable value. On the other hand, the fit indices of the main model such as CFI and IFI are all in an acceptable and appropriate level and the SRMR index is 0.1153.

**Table 3:** Fit indicators of the proposed research model

Desirability	The amount of research findings	Acceptable amount	Indicators
Model approval	4599.861	-	chi-square
Model approval	0.000	-	P-Value
Model approval	2421	*df* ≥ 0	DF(degree of freedom)
Model approval	1.899	*χ*^2^ / *df* < 3	*χ*^2^ / *df*
Model approval	0/061	RMSEA < 0.1	RMSEA
Model approval	0/813	NFI > 0.8	NFI
Model approval	0.899	AGFI> 0.8	AGFI
Model approval	0.899	GFI> 0.8	GFI
Model approval	0.901	CFI > 0.8	CFI
Model approval	0.856	IFI > 0.8	IFI
Model approval	0.1153	.The closer it is to zero	SRMR

## Discussion

The aim of this study was to design an informal payment model to patients admitted in the Iran health system. Based on the data theory model, the foundation was designed as a model in 6 main categories including causal factors, contextual factors, central category (concept of reducing the payment rate), factors affecting the informal the payment, strategies and consequences.

In this study, causal factors included economic factor, socio-cultural factor and poor policy making. Regarding the need to informal of payment in qualitative research, the program of informal the payment is very important, because sustainable national resources, increasing satisfaction with inpatient services in public hospitals and reducing household costs, will improve the quality of health services (25). Due to the necessity of the program, the reduction of the payment rate has led to the reduction of health expenses, the increase of psychological status due to the expansion of insurance coverage and the development of justice in the field of health in the present study, the concept of informal of payment (26). In the present study, the concept of informal payments included out-of-pocket medical expenses paid by the household at the place of receiving services. Reducing the share of OOP payments is one of the main objectives of any health care reform program in the Iran and also the most important emphasis of Iran's health program (27).

Program to reduce the share of informal payments of hospitalized patients in the cost of medical services is one of the duties of governments to the people. Which by implementing it will have many achievements for the health system and increase customer satisfaction (28). Socio-cultural factors have a great impact on the program to informal payments of patients admitted to the health system. Various studies have confirmed the impact of socio-cultural factors on all health policies, introducing these factors with mutual impact. Also, in the other studies, the effect of education level, poverty, intellectual background and medical ethics on the level of public health has been mentioned (29).

In this investigation, underlying and intervening factors including poor management and structure, financial problem, manpower, interventions in strengthening the role of health insurance in eliminating financial communications, interventions in the High Council of Health Insurance and implementation of informal payment program were identified. Regarding the benefits of the reduction plan, reducing people's spending on health care was an effective and appropriate measure that reduced treatment costs in the household basket and prevented many families from falling into poverty (30). Using the annual cost-income survey of households, the results showed that health insurance can play an effective role in reducing inequality in the distribution of medical expenses. Upstream laws require special attention to the development of the insurance system in order to achieve more equal indicators in the distribution of direct payments of household medical expenses, especially in relation to inpatient services (31).

One of the problems of the informal payment program in the present study could be considered as financing and fair distribution of resources. In a cross-sectional retrospective study using the annual household incomeexpenditure survey data provided, the results showed that the fair participation index in financing health expenditures is a symbol of welfare in society and development of the country. This index can provide good evidence for health policy makers to better monitor the effects of health system financing policies, and also the policies adopted in the health sector, including the development of health insurance. The Economic Transformation Plan has failed to achieve the goals of the National Development Plan in justice and financing in the health system (32). Regarding the allocation of human resources in the field of health, the results showed that the distribution of human resources is unfair and the hospitals under study do not follow a specific model or standard in the field of human resource management, and it seems that, bargaining power and influence of managers and the health network is more influential in this matter than any other factor. Therefore, the policy makers of the Ministry of Health and Medical Education should use standard and defined models based on evidence and data to plan in human resources. Use real data to solve the problems and shortcomings of the current unfavorable situation of manpower allocation (33).

In the present study, the strategies included intervention related to the enactment and amendment of the law, intervention in the reform of the tax system, strengthening of cost components and strengthening education. A descriptive-survey study of 30 health insurance experts, revealed that health income (public revenues or designated taxes and public taxes) the value added tax and premium revenues (premiums paid by the government for insured individuals and direct payments of households for health services) were the main and secondary strategies for financing the health insurance plan (34). A qualitative research, indicated that conflict of interest in policy-making is one of the significant issues in basic health insurance package policies and causes conflict of interest and orientation in the formulation of closed policies, so that, there are tendency to expand more the basic health insurance packages and these policies are affected by the specialization and technical field of officials and managers of the Ministry of Health (35). One of the effective factors on informal of payment in the present study with the implementation of the Health Transformation Plan is the increase in induced demand. A study conducted based on the views of experts on informal payment program, showed that induced demand leads to uncontrollable growth of costs, imposition of unnecessary costs and double financial pressure on insurance organizations (23).

In this study, according to the designed model, the implementation of the program to informal of payment in the right way had positive consequences, such as the structural, organizational, monitoring and control. Negative consequences also included information management and document exchange problems, performance deviations, the process and program problems. A qualitative study in the form of content analysis, revealed the positive effects of the program in the form of reducing public payments in the public hospital ward, reducing the number of uninsured people, protecting vulnerable patients and the negative effects of the program in the form of increasing induced demand, increasing total medical costs, increasing the burden of referrals to government centers, increasing the share of health costs in the household budget and increasing the dissatisfaction of medical staff following unfair payments (30).

Regarding discrimination in payments, the experiences of the participants confirm that the implementation of this program will result in injustice and discrimination between physicians and staff, which is consistent with the results of the present study (36).

Regarding the threat to the private sector, the results showed that the implementation of informal the payment program in the health system can be a challenge for private centers, that is consistent with the present study (37). Satisfaction of patients referring to teaching hospitals has decreased compared to before the implementation of the payment reduction program, which is consistent with the present study data (38).

One hundred twenty initial codes, twenty final concepts and six main categories were extracted and the identified categories were placed on the paradigm model by positioning. In this study, according to the identified goals and categories, the category of “ informal the payment of patients admitted” as the main and central category, “economic factor”, “social factor” and “policy making” as the causal factors, “Strengthening Financial Communication”, “Improving the Performance of the High Council of Health Insurance”, and “Developing a Payment Reduction Plan” as interfering factors, “Structural Weakness”, “Financial Problem”, and “Manpower Weakness” as underling factors “Law reform”, “Tax reform”, “Strengthening cost components” and “Strengthening education” as strategic categories, and “Structural improvement”, “Supervision improvement”, and “Information improvement” Were considered as a consequence category. The results of the quantitative ward showed that causal conditions are effective in informal the payment of hospitalized patients. Underlying conditions are effective in informal the payment of hospitalized patients. The interventionist conditions are effective in informal the payment of patients admitted. Underlying factors are effective in the adopted strategy. The strategy adopted is effective in achieving the right outcome.

## Figures and Tables

**Table 1:** Original dimensions, concepts, codes extracted from the perspective of the managers of the Ministry of Health of the Iranian health system

Original dimensions	Concepts	Codes
**Causal factor**	Economic factor	Financial resources, rising medical tariffs, inability of insurance to pay on time and insurance obligations
	Cultural and social	Central justice in the treatment sector, increasing the acceptance of community members from the policies of family physician, empowering people to counter the demand for informal payments, strengthening mechanisms for public participation in the community and health, increasing awareness and transparent flow of information among people
	Poor policy making	Lack of participation of decision-makers and planners in the program. development of policies and regulations, ensuring the implementation of laws, the manner to manage resources and to implement policies
**Axial category**	Imbalance of resources and expenditures	Emphasis on spending resources on treatment, severe recession and reduction of health resources, money management in the health system, relying on disease prevention and reducing treatment costs, strengthening the medical system and the field of discrimination proper management of resources to increase resource effectiveness and lack of proportion of expensive services outside of subsidized basic insurance
	Strengthen of monitoring and control	Quantitative and qualitative monitoring of public insurance and its reimbursement, serious implementation of existing laws by the Health Commission, supervising the registration and certification of patients' payments, increasing insurance supervision in support of the insured with strong legal and financial support, strengthening the feedback system reliable and confidential in payment reports, control of costs of medicines, consumables and equipment , supervision of funds, use of non-governmental and independent institutions in supervision
**Underlying** **factors**	Poor management and structure	Field interventions on structural system reform, serious efforts to implement paragraph 7 of the communicative policies regarding segregation of duties, separation of teaching and non-teaching hospitals, strengthening public insurance funds (efficiency, improving quality and reducing premiums), general policies of the economic system, strengthening the of the health system (accountability, responsibility, transparency, participation, justice and efficiency), centralized organizational structure, separation of non-medical fields from the medical field (training, distribution and supervision by physicians), statistics of the current situation and the current context (opportunities / threats)
	financial problem	Funding, financial protection of patients, fair distribution of resources and credits, fair financial participation and ensuring financial security
	Manpower	Medical system workload, distribution of human resources, presence of experienced physicians in government centers, adequately trained and experienced manpower and distribution of responsible and accountable people appropriate to provide health services in health care system
**Interfering** **factors**	Reinforcement interventions, the role of financial communication	Redefining the relationship of insurance organizations with government providers (no compulsion in performance-based payment contract), increasing the power of insurance supervision in supporting the insured with strong financial and legal support (having legal authority to complain and receive damages) and strengthening the share of basic health insurance, supplementary insurance
	Interventions in the High Council of Health Insurance	Implementation of the same tariff for diagnostic and medical services of the private and public sectors, reform of the structure, composition of members and the arrangement of stakeholders in the Supreme Insurance Council, amendment of legal provisions related to the tariff method of the General Insurance Law and calculation of costs
	Execution of the program	Defining resources, strengthening communication and Between sections cooperation in developing a plan to reduce payments , supporting the program, paying attention to upstream documents, managing the market of drugs, consumables and medical equipment, working closely with basic health insurance, general coverage of health insurance - Strengthening referral system and family physician, uniformity of invoices for hospitalization costs , revision of health service tariffs and revision of the book of relative value of health services and development of monitoring procedures
**Strategies**	Intervention related to the enactment and amendment of the law	Amendment of legal articles and clauses related to the method of tariffs of the General Insurance Law, amendment of tax laws based on unit payment, amendment of the structural process of legislation (Health Commission), determination of transparent and deterrent legal approaches to combat conflict of interest (individual and organizational)
	
	Intervention in tax reform	Preparation of tax return based on individuals' income consolidation card and creating a total income card for individuals and legal entities in the community
	Strengthen of cost components	Strengthening the share of health subsidies , diagnostic services supply chain (referral chain), strengthening the reduction of patients' share, insurance reimbursement, supporting the program to reduce the amount of payment for Paraclinical services, supporting the program to reduce the amount of services, medicine, equipment and supplies from inpatient services (hoteling), support for reducing the amount of payment from clinical services (surgery) supply of medicine, equipment and medical consumables, purchase, distribution , supply and consumption of medical consumables and equipment
	Strengthen of training	Continuous training of hospital financial staff, raising the knowledge of financial staff in the implementation of the program
**Positive** **consequences**	Structural and organizational	More insurance coverage, increased access to medicine, less referral of people out of hospitals for the supply of medicines and medical supplies, reduction of informal patient payments, improving the quality of public hospital hotels, increased patient satisfaction and lack of appropriate organizational structure for health care providers health spending providers
	Monitoring and control	Monitoring direct and indirect payments and not receiving additional pay from patients in the public sector
**Negative** **consequences**	Problem in managing information and exchanging documents	National ID of patients at the time of admission, equipping hospitals with hospital information system (HIS), registration of transaction ID and electronic health record ID, creating a national health network or national internet service, collecting accurate and timely information from organizations providing health services
	Performance deviation	Increased medical expenses, rising inflation, deviation in the purpose of health subsidies and lack of insurance mechanisms appropriate to the implementation of the program
	Process and planning problem	Discrimination in payments, threats to the private sector, induction of demand in health care providers and recipients, increased expectations of health clients, increased inflation in the health sector, bankruptcy of insurance companies, imposing heavy costs on the government, increasing workload for non-medical staff , creating income gap in physicians and other staff , dissatisfaction of other staff such as nurses with adverse consequences, inefficient health system, inadequate structure design, applying rapid vertical corrections in the health system, lack of investment and lack of strengthening components.

## References

[1] McKee M, Balabanova D, Basu S, Ricciardi W, Stuckler D (2013). Universal Health Coverage: A Quest for All Countries But under Threat in Some. Value in Health.

[2] Hummy Song H, Tucker A (2016). Performance Improvement in Health Care Organizations. Technology Information and Operations Management.

[3] Yu CP, Whynes DK, Sach TH (2008). Equity in health care financing: the case of Malaysia. Int J Equity Health.

[4] Kolasa K, Kowalczyk M (2016). Does cost sharing do more harm or more good?-a systematic literature review. BMC Public Health.

[5] Ekman B (2007). The impact of health insurance on outpatient utilization and expenditure: evidence from one middle-income country using national household survey data. Health Res Policy Sys.

[6] Piroozi B, Rashidian A, Moradi G, Takian A, Ghasri H, Ghadimi T (2017). Out-of-Pocket and Informal Payment Before and After the Health Transformation Plan in Iran: Evidence from Hospitals Located in Kurdistan, Iran. Int J Health Policy Manag.

[7] Arsenijevic J, Pavlova M, Groot W (2015). Out-of-pocket payments for health care in Serbia. Health Policy.

[8] Carrin G, Mathauer I, Xu K, Evans DB (2008). Universal coverage of health services: tailoring its implementation. Bull World Health Organ.

[9] Chereches RM, Ungureanu MI, Sandu P, Rus IA (2013). Defining informal payments in healthcare: a systematic review. Health Policy.

[10] European Commission (2013). Study on Corruption in the Healthcare Sector: Office of the European Union.

[11] RM, Ungureanu MI, Rus I, Baba C (2011). Informal Payments in the Health Care System - Research, Media and Policy. Transylvanian Review of Administrative Sciences.

[12] Parsa M, Aramesh K, Nedjat S, Kandi MJ, Larijani B (2015). Informal Payments for Health Care in Iran: Results of a Qualitative Study. Iran J Public Health.

[13] Nekoeimoghadam M, Esfandiari A, Ramezani F, Amiresmaili M (2013). Informal payments in healthcare: a case study of kerman province in Iran. Int J Health Policy Manag.

[14] Vafaei Najar A, Ebrahimipour H, Pourtaleb A, Esmaily H, Jafari M, Nejatzadegan Z, Molavi Taleghani Y (2017). At first glance, informal payments experience on track: why accept or refuse? Patients perceive in cardiac surgery department of public hospitals. northeast of Iran 2013. BMC Health Serv Res.

[15] Aboutorabi A, Ghiasipour M, Rezapour A, Pourreza A, Sarabi Asiabar A, Tanoomand A (2016). Factors affecting the informal payments in public and teaching hospitals. Med J Islam Repub Iran.

[16] Zarei E, Palesh M, Khodakarim S, Nikkhah A (2018). Informal Payments for Inpatient Services and Related Factors: A Cross-Sectional Study in Tehran, Iran. Health Scope.

[17] Khodamoradi A, Rashidian A, Aghlmand S, Arab M, Moini M (2015). Informal payments and its related factors in Urmia hospitals. Hakim Health Sys Res.

[18] Meskarpour-Amiri M, Arani AA, Sadeghi H, Agheli-Kohnehshahri L (2016). Socioeconomic factors affecting informal payments in the health sector. Transylvanian Rev Administ Sci.

[19] Mirabedini SA, Fazl Hashemi SME, Sarabi Asiabar A, Rezapour A, Azami-Aghdash S, Hosseini Amnab H (2017). Out-of-pocket and informal payments in Iran's health care system: A systematic review and meta-analysis. Med J Islam Repub Iran.

[20] Vahidi R, Saadati M (2013). Determining the distribution of effective factors on out of pocket payment (formal and informal) in hospitalized cardiac patients of Shahid Madani hospital and its side effects on the patient or companions-Iran-Tabriz 2010. Hospital Journal.

[21] Vian T, Burak LJ (2006). Beliefs about informal payments in Albania. Health Policy Plan.

[22] Miller K, Vian T (2010). Strategies for reducing informal payments.

[23] Moradi-Lakeh M, Vosoogh-Moghaddam A (2015). Health Sector Evolution Plan in Iran; Equity and Sustainability Concerns. Int J Health Policy Manag.

[24] Golinowska S (2010). Informal payments in health care. Polish perspective and experience. Zeszytu Naukowe Ochrony Zdrowia Zdrowie Publiczne i Zarządzanie.

[25] Yusefi AR, Bastani P, Bordbar S, Sadeghi A, Hesami SZ (2018). The Effects of Health Transformation Plan Implementation on the Performance Indicators of Public Hospitals. Health Scope.

[26] Mousavi SM, Sadeghifar J (2016). Universal health coverage. The lancet global health.

[27] Akhondzade R (2014). Health system transformation project. an opportunity or a threat for doctors (Editorial). JAP.

[28] Berman PA (1998). Rethinking health care systems: private health care provision in India. World Development.

[29] Alahmad G, Al-Jumah M, Dierickx K (2012). Review of national research ethics regulations and guidelines in Middle Eastern Arab countries. BMC medical ethics.

[30] Peikanpour M, Esmaeli S, Yousefi N, Aryaeinezhad A, Rasekh H (2018). A review of achievements and challenges of Iran's health transformation plan. Payesh.

[31] Khammarnia M, Barfar E, Ansari-Moghadam A, Setoodehzadeh F, Zanganeh Baygi M (2018). The Households Health Spending and Impoverishment: A Study After Iran's Health Transformation Plan. Health Scope.

[32] Parsa M, Aramesh K, Nedjat S, Kandi MJ, Larijani B (2015). Informal payments for health care in Iran: results of a qualita¬ tive study. Iran J Public Health.

[33] Adham D, Mahdavi A, Mehrtak M, Ebrahimi K, Azari A (2016). Assessment of Human Resource Allocation in General University Hospitals in the Cities of East Azerbaijan Province. J health.

[34] Davari M, Haycox A, Walley T (2012). Health Insurance system in Iran; past experiences, present challenges and future strategies. Iranian J Publ Health.

[35] Mohammadi E, Olyaee Manesh A, Rashidian A, Hasanzadeh A, Razavi M (2020). Policy Analysis, Problem Identification and Proposing Policy Options for Health Insurance Benefit Package in Iran. BMC Health Service Re.

[36] Meng R, Li J, Zhang Y, Yu Y, Luo Y, Liu X, Zhao Y, Hao Y, Hu Y, Yu C (2018). Evaluation of Patient and Medical Staff Satisfaction regarding Healthcare Services in Wuhan Public Hospitals. Public Health.

[37] Thompson R, Witter S (2000). Informal payments in transitional economies: implications for health sector reform. Int J Health Plann Manag.

[38] Shariati A, Jamshid Beigi Y, Baraz Pardnjati Sh, Haghighizadeh MH, Abbasi M (2017). Assessment of nurses, patient satisfaction, patient attendants in educational hospitals in Ahvaz city health development plan in 2015. Journal of Clinical Nursing and Midwifery.

